# Transcriptomic Analysis of the *Pistacia vera* (L.) Fruits Enable the Identification of Genes and Hormone-Related Gene Linked to Inflorescence Bud Abscission

**DOI:** 10.3390/genes13010060

**Published:** 2021-12-27

**Authors:** Jubina Benny, Antonio Giovino, Francesco Paolo Marra, Bipin Balan, Federico Martinelli, Tiziano Caruso, Annalisa Marchese

**Affiliations:** 1 Department of Agricultural, Food and Forest Sciences, University of Palermo, Viale delle Scienze—Ed. 4, 90128 Palermo, Italy; jubina.benny@unipa.it (J.B.); bipin.balan@unipa.it (B.B.); tiziano.caruso@unipa.it (T.C.); annalisa.marchese@unipa.it (A.M.); 2 Council for Agricultural Research and Economics (CREA), Research Centre for Plant Protection and Certification (CREA-DC), 90011 Bagheria, Italy; antonio.giovino@crea.gov.it; 3 Department of Architecture (DARCH), University of Palermo, Viale delle Scienze—Ed. 8, 90128 Palermo, Italy; 4 Department of Biology, University of Florence, Sesto Fiorentino, 50019 Florence, Italy; federico.martinelli@unifi.it

**Keywords:** *Pistacia vera*, alternate bearing, differentially expressed genes DEGs, hormones, flower bud abscission

## Abstract

*Pistacia vera* (L.) is an alternate bearing species. The tree produces axillary inflorescence buds every year. Still, they abscise in “ON” overloaded shoots, causing a limited production in the following “OFF” year, causing a significant and unfavorable production fluctuation. In this work, we carried out de novo discovery and transcriptomic analysis in fruits of “ON” and “OFF” shoots of the cultivar Bianca. We also investigated whether the fruit signaling pathway and hormone biosynthesis directly or indirectly linked to the premature fall of the inflorescence buds causing alternate bearing. We identified 1536 differentially expressed genes (DEGs) in fruits of “ON” vs. “OFF” shoots, which are involved primarily in sugar metabolism, plant hormone pathways and transcription factors. The premature bud abscission linked to the phenomenon is attributable to a lack of nutrients (primarily sugar) and the possible competition between the same branches’ sinks (fruits vs. inflorescence buds). Hormone pathways are involved as a response to signals degradation and remobilization of carbon and nutrients due to the strengthening of the developing embryos. Genes of the secondary metabolism and transcription factors are also involved in tailoring the individual branches response to the nutritional stress and sink competition. Crosstalk among sugar and various hormone-related genes, e.g., ethylene, auxin, ABA and cytokinin, were determined. The discovery of putative biomarkers like *callose synthase 5*, *trehalose-6-phosphate synthase*, *NAD(P)-linked oxidoreductase* and *MIOX2*, Jasmonate, and salicylic acid-related genes can help to design precision farming practices to mitigate the alternate bearing phenomenon to increase farming profitability. The aim of the analysis is to provide insight into the gene expression profiling of the fate of “ON” and “OFF” fruits associated with the alternate bearing in the pistachio.

## 1. Introduction

*Pistacia vera* (L.) is a xerophytic, deciduous tree native of central and west Asia arid regions, including Iran and Afghanistan. It is a species with unusual alternate bearing behavior. The plant carries out all the main vegetative and reproductive phases in a short period, between the mid of March and the end of May, in the mature pistachio trees. The current season’s shoot’s growth pattern is exclusively dominant, and it extends from the vegetative terminal bud of the previous season’s shoot. A single axillary bud is present under each compound leaf on the current season’s growth. Most axillary buds differentiate into inflorescence primordia; therefore, flowering and fruit production occurs on 1-year-old wood. The tree differentiates axillary inflorescence buds every year, but they abscise in “ON” year with massive production, causing a limited production in the following “OFF” year [1,2,3,4,5,6]. To simplify this phenomenon by outlining a timeline, lower buds start to abscise or drop at the end of June and continue in July and August, determining the heavy reduction in production in the next year, thus resulting in an “OFF” year. As the inflorescence bud abscission happens during the active growth and expansion of the embryo (July–August), the phenomenon has been related to the competition for carbohydrates between sink fruits on the previous year’s wood versus inflorescence buds on the current year’s shoots [2,3,7,8,9]. At the same time, some studies proved that hormone concentrations are also involved in inflorescence bud abscission [6]. Auxin, Cytokinin, and polyamines may significantly affect the abscission of pistachio flower buds [10,11].

Many transcriptomic studies have been carried out to understand better the alternate bearing phenomenon at the molecular level in many species. For example, studies proved that inhibition of genes like *Flowering locus T* (*FT*), *LEAFY* (*LFY*), *Suppressor of overexpression of constans 1* (*SOC1*) and *APETALA1* (*AP1*) during heavy crop act as a key factor for the alternate bearing in *Arabidopsis thaliana* and *mandarin* [12,13,14]. At the same time, genes like *Terminal flower 1* (*TFL1*), *Flowering locus C* (*FLC*), *Brother of FT* (*BFT*), and *Short vegetative* phase (*SVP*) were involved in the floral enhancement in *Arabidopsis* [15]. In contrast, in *Malus domestica*, gibberellin related genes are more likely to be engaged in alternate bearing than flowering genes [16].

In pistachio, a recent experiment of high-throughput RNA-Seq analysis has been performed by Benny et al. (2020) [11] on inflorescence buds of “ON” and “OFF” trees of the Italian cultivar Bianca and provided insight into the mechanism leading to the premature inflorescence bud abscission [11]. From this study, it is evident that the lack of resources (primarily carbohydrates) triggers a cascade of events that lead to the fall of the buds of the loaded “ON” branches starting around the third week of June [11], indicating a source-sink competition. Inflorescence bud abscission can be considered a “within shoot phenomenon” interconnected to the crop load on the one-year-old growth.

In this study, the inflorescence buds of “ON” branches in June and July showed an intense starch degradation due to the up-regulation of *ALPHA-AMYLASE 3*, over-expression of genes involved in the biosynthetic pathways of raffinose and of the *MIOX* gene, which are activated in conditions of limited energy reserves and stress. Changes in carbohydrate levels affected the trehalose-6-phosphate, which was found down-regulated [11], involved in the genetic control of sucrose levels and sink development [17]. The *SnRK1* gene key regulator of the plant energy balance and involved in the stress signaling network, which includes the *TOR kinase* [18], was given as active. The *auxin-mediated TOR* gene, which regulates translation, transcription, cell growth and proliferation, differentiation, and autophagy [19] was also affected, confirming its involvement in various signal responses, including nutrient availability, stress, and hormones [11,20]. At the hormonal level, the genes involved in the biosynthetic pathways of auxins were down-regulated, and the polyamines (PA), including spermidine, were down-regulated. The reason might be the rapid degradation of PA in the apoplast with high hydrogen peroxide (H_2_O_2_) production that activates ROS-dependent oxidative stress mechanisms. This eventually induces autophagy and programmed cell death, leading to inflorescence bud abscission, together with the action of the *TOR* gene [11].

A preliminary study comparing fruits of “ON” and “OFF” trees showed that genes related to photosynthesis, α-amylase, and terpenoid were up-regulated in “ON” trees [21]. From these two studies, the signal cascades and the metabolic pathways involved gave clues to a possible crosstalk among fruit and bud, likely mediated by hormones. The present study was undertaken to complete the previous analysis conducted by Benny et al. (2020) [11] to investigate the fruit signaling pathway and putative crosstalk by RNA-Seq analysis, carried out in fruits of “ON” and “OFF” shoots of the cultivar Bianca for a deeper understanding of the complex molecular network underpinning the premature inflorescence bud abscission process. This study helped us to identify the presence of inhibitory signals or genes in fruits related to hormone biosynthesis directly or indirectly linked to the premature fall of the inflorescence buds, considered the leading cause of alternate bearing behavior in *Pistacia vera* (L.).

## 2. Materials and Methods

### 2.1. Sample Collection, Sequencing, and Pre-Processing

The *Pistacia vera* (L.) fruit samples were collected for the transcriptomics analysis on the 27 June (61 days after full boom (DAFB)) and on 22 July 2019 (86 DAFB) from the “Fratelli Morello” (N 37°26′3.192″, E 14°3′11.988″) farm in the inland of Sicily (37°30′ Lat. N) (Figure S1). Three Bearing branches (“ON”) contained around 50 fruits, and three non-bearing branches (“OFF”) with a maximum of 8 fruits were tagged after the fruit set (Figure S2). Three biological replicates from the same tree were collected from “ON” and “OFF” branches of June and July, which eventually composed 12 samples.

Therefore, 3–6 fruit tissues were collected from each sample of both bearing and non-bearing branches. From now on we refer to them as “ON” fruits and “OFF” fruits, respectively. After the tissue collection, all the samples were immediately frozen in the liquid nitrogen and stored at −80 °C. Using the Spectrum Plant Total RNA Kit (Sigma-Aldrich), the total RNA of 100 mg from the grounded sample was extracted. Agilent Bioanalyzer was used to check the RNA quality and RNA Integrity Number (RIN). TruSeq RNA-Seq sample prep kit from Illumina (Illumina, Inc., San Diego, CA, USA) was used to obtain the sample libraries. The sequencing was done by BMR Genomics (Padua, Italy) using ultra-high throughput on the Illumina HiSeq 2000 (Illumina Inc., San Diego, CA, USA) to obtain single reads per sample, each 75 bp long.

### 2.2. Transcriptomic Analysis, Annotation, and Evaluation

FastQC (https://www.bioinformatics.babraham.ac.uk/projects/fastqc/, accessed on 19 November 2021) (version 1.16) was used to identify the quality of the raw data generated from the sequencing machine. Based on the results from the FastQC, the low-quality reads and the adapters were removed using a custom-made Perl script and cutadapt version 2.0 (Table S1). To remove all the rRNA reads and to obtain clean reads, the pre-processed reads were aligned against the Silva (https://www.arb-silva.de/, accessed on 19 November 2021) database using the bowtie [22] aligner (version 2.3.4.1). Then, using the default parameters of the Trinity (version 2.8.4) assembler, the clean reads from 12 pre-processed samples were assembled. A clustering threshold of 98% identity was used in CD-Hit-EST [23] (version 4.6.8) for obtaining the transcript clustering to reduce the redundancy. The transrate (http://hibberdlab.com/transrate/, accessed on 19 November 2021) program was used to collect the assembly statistics. Using the plantae dataset (viridiplantae_odb10), the results from the assembly was evaluated by BUSCO [24] (version 3.0.2), which is a tool for assessing genome completeness based on the existence of single-copy orthologs (Table S2). The raw data from the sequencing produced around 227 and 234 million raw reads as a single-end, respectively, for the June and July tissue collection. Therefore, only the reads with an average Phred quality score of 38 were selected for the downstream assembly analysis.

The overall assembly and evaluation results are given in Table S2. The workflow used for the de novo assembly, discovery, and annotation are provided in Figure S3.

The annotation was done using BLASTx (http://www.ncbi.nlm.nih.gov/BLAST/, accessed on 19 November 2021) program for the obtained contigs using an E-value threshold of 1e−5 against NCBI “nr” database (https://www.ncbi.nlm.nih.gov/refseq/about/nonredundantproteins, accessed on 19 November 2021), PFAM database (https://pfam.xfam.org, accessed on 19 November 2021), InterPro database (https://www.ebi.ac.uk/interpro/, accessed on 19 November 2021), UniProt protein database (https://www.uniprot.org, accessed on 19 November 2021), STRING database (https://string-db.org, accessed on 19 November 2021), and KEGG database (http://www.genome.jp/kegg, accessed on 19 November 2021). Only the contigs aligned to ‘Viridiplantae’ and ‘unannotated’ were considered for the final transcriptome assembly. The raw RNA-Seq data for this work can be accessed through NCBI’s SRA under accession number PRJNA623387.

### 2.3. Differentially Expressed Genes (DEGs) between “ON” and “OFF”

To estimate the gene expression levels from RNA-Seq data, we used RSEM [25]. The output from the RSEM is an expected count matrix which is given as the input for edgeR [26]. The comparison among the stages selected for the study is given in Table 1. The genes with adjusted *P*-value (FDR) lower than 0.01 and at least a two-fold change were considered as significantly differentially expressed genes in the samples. Using the p.adjust function of R, all the statistical tests were corrected for multiple comparisons using the Benjamini–Hochberg false discovery rate. This approach can make the FDR at the desired level of α (in this study 0.01) by adjusting the *P*-values. R software was used for the statistical analysis. Differences among the selected samples were adjusted using the sample normalization. To remove systematic variation between replicates, the normalization procedure served as a crucial pre-processing step to adjust for the different sample sequencing depths and other confounding technical effects. We used the geometric normalization method where FPKMs and fragment counts are scaled via the median of the geometric means of fragment counts across all libraries, as described in [27]. The clustering dendrogram plot was generated for identifying the clustering patterns of the samples (Figure S4). The grouping of the clusters for dendrogram was done using the Euclidean distance measure. At the same time, to identify the GO terms and metabolic pathways that were significantly enriched in DEGs, functional-enrichment analysis was performed.

### 2.4. Functional and Gene Enrichment Analysis

To get the corresponding TAIR ID, the final contigs were aligned against TAIR10 (https://www.arabidopsis.org) sequence using the blastx program. Finally, a mapping file for pistachio was generated using the blastx results. There were five categories generated for the mapping file:

(1) Nearly identical: Score ≥ 1000 and e-value = 0.
(2) Highly similar: Score ≥ 1000 and e-value ≠ 0 OR (Score ≥ 500 & Score < 1000) and e-value = 0.
(3) Moderately similar: (Score ≥ 200 & Score < 1000) and e-value ≠ 0.
(4) Weakly similar: (Score ≠ 100 & score < 200).
(5) Very weakly similar: (Score < 100) based on the blastx score and e-value.

The pistachio mapping file is available at https://drive.google.com/open?id=1nMp2euy36JwVtXIrVibEdnkaVB_AV2Uv (accessed on 19 November 2021).

To map and visualize the gene IDs and the hormone regulation, CHO metabolism, metabolic overview, secondary metabolism, and transcription factors, we used MapMan [28]. We used two files for the visualization and mapping: (1) results related to “OFF” and “ON” stages of fruit, and (2) results comparing the time-point (June and July).

To visualize the differences between metabolic pathways using the parameters like over-representation analysis (ORA) cutoff value of 3, Wilcoxon tests, and no correction, the PageMan analysis plugin of MapMan was used. For the PageMan analysis, we considered all the DEGs present related to comparing “ON” and “OFF”, June and July. The DAVID (Database for Annotation, Visualization, and Integrated Discovery) version 6.8 [29] Web server (https://david.ncifcrf.gov/, accessed on 19 November 2021) was used to extract the gene ontology related information.

## 3. Results

### 3.1. Transcriptomic Assembly and Annotation Results

The details explaining the N50 length, transcripts mean length, BUSCO scores, and percentage of the alignment for the pre-processed reads were given in Table S2.

RSEM was used for the quantification of the genes. The count matrix generated by RSEM was then taken as the input for the edgeR tool. A blastx search was done against different databases like PFAM, KEGG, InterPro, STRING, Uniprot/Swissprot, and non-redundant (NR) protein database for annotating the assembled transcripts. Our first sampling was made during the third week of June (61 DAFB) as it is likely for the cv. Bianca to face a limitation of resources during this period. Moreover, it reached a maximum peak in a month when we did the second sampling of the plant material (86 DAFB). The percentage of inflorescence bud drop was strongly affected by the crop load. Bud drop resulted absent in June OFF branches and negligible in ON ones, whereas in July, ON branches showed more than double the percentage of bud drop compared with the OFF ones (Table 1)

Table 1 reports the values of inflorescence bud drops in “ON” and “OFF” branches in June, July, and September. In June (coincident with the first sampling period), the inflorescence bud drop value was 7.4% in ON branches, in July (coincident with the second sampling period) it reached 54.7% in ON branches, in September it was 78.3% in ON branches, while the OFF branches showed a very different trend showed (0% bud drop in June, 25.4% in July, and 31% in September).

In addition, the number of enhanced and repressed genes and the total number of genes obtained in each sample comparison are listed in Table 2.

### 3.2. Effect of Crop Load on Photosynthesis in Fruits of “OFF” vs. “ON” Branches

The gene expression in fruits of “ON” branches showed an enhancement of photosynthetic activity. Most of the genes involved in photosynthesis were down-regulated in fruits of “OFF” branches. The photosystem II PSII polypeptide subunit and photosystem II LHC-II subunits (*CHLOROPHYLL PROTEIN 24*, *LIGHT-HARVESTING CHLOROPHYLL B-BINDING 2* and *light-harvesting complex gene 1*) were down-regulated in “OFF” fruit. In contrast, a gene calling for photorespiration, *D-isomer specific 2-hydroxy acid dehydrogenase*, and *ATP synthase* (*PIGMENT DEFECTIVE 332*) were enhanced during the “OFF” period. The genes encoding for the Calvin cycle *ribulose bisphosphate carboxylase* and *transketolase 2* were repressed in “OFF” period (Table S3).

### 3.3. Effect of Crop Load on Starch Metabolism in Fruits of “OFF” vs. “ON” Branches

**Figure 1:**
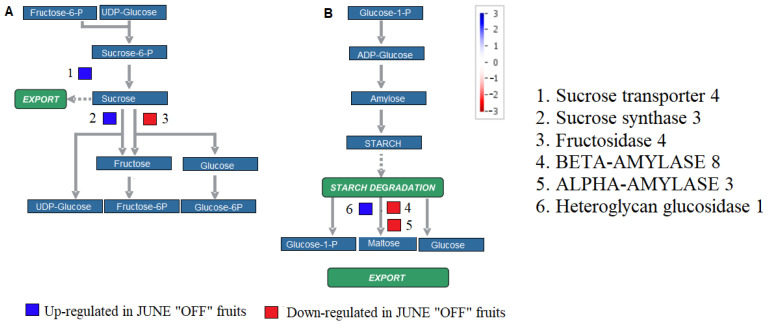
The figure shows the MapMan pathways in sucrose-starch metabolism. The figure highlights differentially expressed genes between fruits in the non-bearing shoot (June “OFF”) and bearing shoot (June “ON”) in sucrose degradation (**A**) and starch synthesis (**B**) pathways. Small squares represented individual genes. The color scale indicates the log2 FC value. Red represents down-regulation, and blue represents up-regulation in June “OFF” relative to June “ON”.

The study on the relationship between crop load and starch metabolism help in assessing the functional distribution of starch in “ON” and “OFF” fruits. The genes encoding for *sucrose transporter 4*, *sucrose synthase 3*, and *heteroglycan glucosidase 1* were enhanced in the fruit tissue of the “OFF” branches. On the other hand, the genes encoded for *ALPHA-AMYLASE 3*, *BETA-AMYLASE 8*, and *fructosidase 4*, involved in starch degradation, were repressed in “OFF” fruits, and enhanced in “ON” fruits (Figure 1).

### 3.4. Effect of Crop Load Status on Transcription Factors in Fruits of “OFF” vs. “ON” Branches

**Figure 2:**
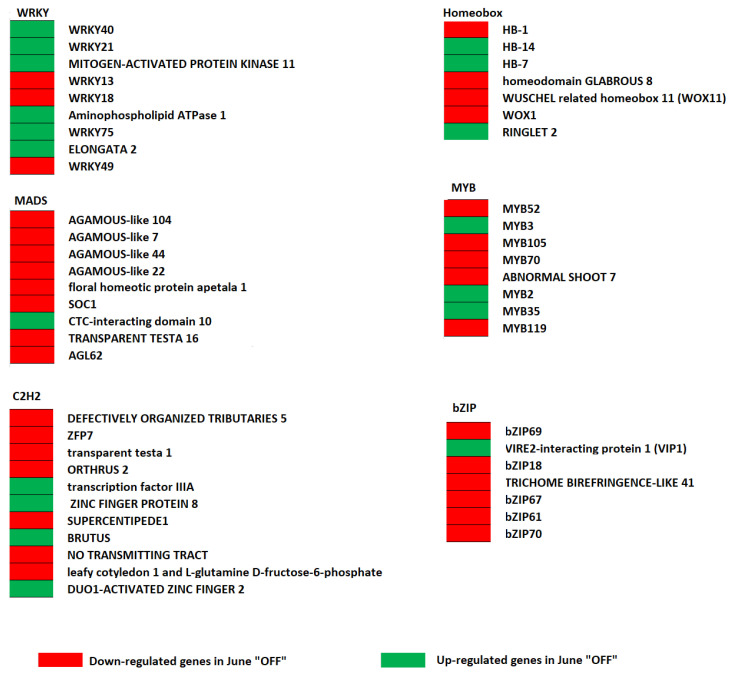
The figure shows transcription factors among the June “OFF” vs. June “ON” fruits comparison. Red and green represents down-regulated and up-regulated genes in June “OFF”, respectively.

In June “OFF” fruits, genes related to *bZIP* (*bZIP18*, *bZIP61*, *bZIP67*, *bZIP70,* and *trichome birefringence-like 41*), *WRKY13*, two homeobox genes (WUSCHEL-related homeobox 11 (WOX11) and *homeodomain GLABROUS* 8) were down-regulated (Figure 2). In June “OFF”, *C2H2* factors (zinc finger protein 7, *transparent testa 1*, *leafy cotyledon 1,* and *L-glutamine D-fructose-6-phosphate*), *MADS* factors (*AGAMOUS-like 104* and floral *homeotic protein apetala 1),* and *MYB* factors (*MYB52, Abnormal shoot 7*, *MYB119,* and *MYB70*) were also down-regulated (Figure 2). In June “OFF”, *WRKY* factor (*WRKY40*, *WRKY*75, *ELONGATA 2*, mitogen-activated protein kinase, and Amino phospholipid ATPase 1) and three *MYB*-related genes (*MYB3*, *MYB2,* and *MYB35*) were up-regulated (Figure 2).

Meanwhile, in July “ON”, four *WRKY* factors (*WRKY75*, *ABA-overly sensitive 1*, mitogen-activated protein kinase, and UDP-glycosyltransferase) and two Aux/IAA-related genes (*argonaute 5* and *indole-3-acetic acid-inducible 30*) were up-regulated (Figure S5). Furthermore, in July “ON” fruits, the study reported enhancing *APETALA2* (*ethylene and salt inducible 3*, *ARIA-interacting double AP2 domain protein*, *WRINKLED 1*) and *MADS* factors like *AGAMOUS-like 104* and *floral homeotic protein apetala 1*.

### 3.5. Effect of Crop Load on Hormone Metabolism in Fruits of “OFF” vs. “ON” Branches

**Figure 3:**
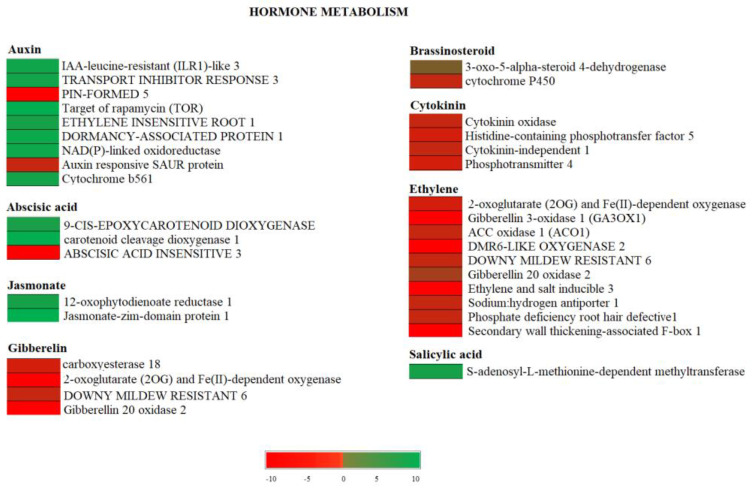
The figure shows hormone metabolism in Pistachio among the June “OFF” vs. June “ON” fruits comparison. The color scale indicates the log2 FC value. Red represents down-regulation and green represents up-regulation in June “OFF” fruits relative to June “ON” fruits.

The genes involved in hormone-related categories are summarized in Figure 3.

Repression of ethylene, gibberellin, and cytokinin pathways were identified in June “OFF” fruits, whereas ABA, IAA, and Jasmonate pathways mainly were up-regulated. In June “OFF” fruits, all the genes responsive to ethylene, gibberellin, brassinosteroid, and cytokinin were down-regulated. Relating to auxin-responsive genes, down-regulation of *PIN formed 1* and *SAUR* and the up-regulation of the *target of rapamycin* (*TOR*), *Ethylene insensitive root 1*, and *cytochrome B561* were observed. Relating to ABA, there was a down-regulation in *abscisic acid insensitive 3* and up-regulation in *Carotenoid cleavage dioxygenase 1* and *9-cis-epoxycarotenoid dioxygenase*. In addition, several genes involved in ethylene biosynthesis and signalling such as *2-oxoglutarate* (*2OG*) and *Fe (II)-dependent oxygenase*, *Gibberellin 3-oxidase 1* (*GA3OX1*), *ACC oxidase 1* (*ACO1*), *DOWNY mildew resistant 6* and *phosphate deficiency root hair defective 1* were repressed during in “OFF” fruits. We also observed an up-regulation in *S-adenosyl-L-methionine-dependent methyltransferase* related to Salicylic acid (Figure 3). Similar expression profiles were found in July “OFF” fruits (Figure S6).

### 3.6. Effect of Crop Load Status on Polyamines in Fruits of “OFF” vs. “ON” Branches

The gene expressed in “OFF” fruits exhibited an enhancement of polyamines (PA) and spermidine (Spd) than the “ON” fruits (Figure S7). In “OFF” fruits, the genes encoding for *thermospermine synthases* (*ACL5*), probable *polyamine transporter*, and *spermidine synthase 1* (*SpeE*) were enhanced. On the contrary, the expression of *S-adenosyl methionine carrier 2* was repressed.

### 3.7. Effect of Crop Load on Carbohydrate Metabolism and Mobilization in Fruits of “OFF” vs. “ON” Branches

**Figure 4:**
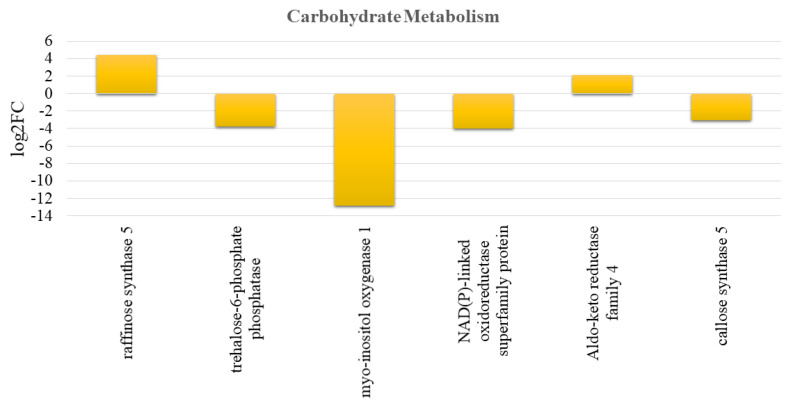
The figure shows the carbohydrate metabolism and mobilization pathway in pistachio among the June “OFF” vs. June “ON” fruits comparison. The y-axis represents the value of log2 fold change. The bar indicates the effects on carbohydrate levels driven by differential expression of different CHO metabolism genes.

The relationships between the carbohydrate metabolism and mobilization pathway in Pistachio fruit from non-bearing branches (June “OFF”) and bearing shoots (June “ON”) are indicated in Figure 4.

*Raffinose synthase* gene (*Raffinose synthase 5* (*RS5*) and Aldo-keto reductase family 4 were enhanced in June “OFF” fruits. On the other hand, sugar alcohols such as *callose synthase 5*, *trehalose-6-phosphate synthase*, *NAD(P)-linked oxidoreductase* and *MIOX2* were repressed in the “OFF” fruits (Figure 4). The comparative study on starch metabolism of the July “OFF” vs. July “ON” fruits produced similar results to the results of June “OFF” vs. June “ON” fruits (Figure S8).

## 4. Discussion

The development of genomic and transcriptomic studies has contributed to a better understanding of the molecular and physiological processes involved in the bud abscission phenomenon in the pistachio tree. Our recent transcriptomic experiment by Benny et al. (2020) [11] on inflorescence buds of “ON” and “OFF” branches of the cultivar Bianca showed that the lack of resources (primarily carbohydrates) was the leading cause of inflorescence bud abscission in “ON” branches, indicating the “branch semi-autonomy” [11]. Inflorescence bud abscission in the pistachio seems a “within shoot phenomenon” interconnected to the crop load on the one-year-old growth. This is also supported by the observations on a slight inflorescence bud drop during the season even in non-bearing pistachio branches of “ON” trees. For instance, in “ON” branches in June (coincident with the first sampling period) the inflorescence bud drop was 7.4%, then it intensified in July (coincident with the second sampling period) reaching a value of 54.7%, while a very different trend was shown in the “OFF” branches with less than eight fruits (0% bud drop in June and 25.4% in July). Severe inflorescence bud drop on fruiting branches is significantly correlated to embryo/fruit development stages and sink competitions for nutrients within the same branch [30].

Previous studies on the role of carbohydrate in inflorescence bud abscission showed that the fruit is dominant in competing for photosynthates compared to inflorescence buds in pistachio [31]. Inflorescence bud abscission in pistachio occurs due to the deficiency of carbohydrates transferring from the adjacent leaves [1,31]. In the present RNA-Seq analysis, genes *ALPHA-AMYLASE 3*, *BETA-AMYLASE 8* and *fructosidase 4*, *callose synthase 5*, *trehalose-6-phosphate synthase* (*T6P*), *NAD(P)-linked oxidoreductase,* and *MIOX2* were up-regulated in both June and July “ON” fruits, and down-regulated in “OFF” fruits. Among hydrolases, it has been demonstrated that alpha-amylases have a central role in reserve starch mobilization to support seedling growth. *Alpha-amylase* expression is induced by hormone gibberellin (GA) and sugar demand/starvation [32]. According to our model [11], sugars availability regulate organ developmental, interacting with nutrient signaling sensor, like *trehalose-6-phosphate gene* (*T6P*) and *SnRK1* (*SUCROSE-NON-FERMENTING-1-RELATED PROTEIN KINASE-1* gene).

T6P can be considered an indicator of sucrose status in plants. It is a crucial sugar signaling that plays a pivotal role in regulating sugar metabolism; it correlates with higher levels of soluble carbohydrate levels and increased photosynthesis capacity through ABA signaling [33]. There is a regulatory loop, which involves *T6P*, *SnRK1* (negatively regulated by *T6P*), and *bZIP* transcription factors that control sucrose availability and utilization [34,35]. In the present study, “ON” fruits showed an enhancement of the *T6P* gene and down-regulation of *SnRK1*, which is opposite to the expression pattern in “ON” buds found by Benny et al. (2020) [11], demonstrating the strength of fruit as the strongest sink and the competition for carbohydrates exerted by the fruits. Furthermore, it has been shown that the nutrient starvation signaling gene, *SnRK1* synergistically with ABA modulates source-sink communication in cereal seedlings under abiotic stress [33,36]. A similar scenario possibly is acting in pistachio. *MIOX* and *NAD(P)-linked oxidoreductase* genes were found also up-regulated in inflorescence buds of “ON” branches. Therefore, it is evident that signaling of nutritional stress are already manifesting in pistachio “ON” branches in June and July, as well as protective mechanism against oxidative burst, showing the semi-autonomy of the branches. In *Arabidopsis thaliana* and tomato, up-regulation of the *myo-inositol oxygenase* (*MIOX*) gene occurs under sugar starvation conditions to generate alternative sugar sources, thereby indirectly contributing to metabolic homeostasis [37,38], and in ascorbate biosynthesis, for scavenging reactive oxygen species [38]. In transgenic sweet potato under stress, over expression of *MIOX* induces higher proline and trehalose production and increased photosynthesis capacity [39]. Studies on Arabidopsis mutants provide evidence that changes in *NAD(P)-linked oxidoreductase* status can alter photosynthesis and plant stress responses [40,41].

A system for scavenging ROS is active in the ON fruits, mediate by hormone signaling (ABA), the antagonistic *T6P* and *SnRK1* regulatory loop and the gene expression rate of transcription factors (like *WRINKLED1* (*WRI1*), *APETALA2* (*AP2*), *MADS factors AGAMOUS-like 104*). Meanwhile, in inflorescence buds in “ON” branches, a ROS mediated programmed cell death process takes place, presumably linked to the over-expression of *SnRK1* which inhibits vegetative growth under energy stress, as shown in the model proposed by Benny et al. 2020 [11]. Interestingly, ethylene-responsive genes and transcription factors regulating the expression of genes involved in the allocation of carbon into oil are up-regulated in “ON” fruits, confirming their strength as a sink. Indeed, in July “ON” fruits, *APETALA2* (a member of the AP2/EREBP ethylene-responsive element-binding protein [42]), *WRINKLED 1*, and *MADS* factors like *AGAMOUS-like 104* were enhanced. *WRINKLED1* (*WRI1*, AT3G54320) is an APETALA2/ethylene-responsive element-binding protein (AP2/EREBP) transcription factor regulating the expression of genes involved in carbon allocation into oil or triacylglycerol (TAG) in plants [43,44,45]. The turnover rate of *WRI1* is influenced by *SnRK*1, which controls the proteasomal degradation of *WRI1* by phosphorylating its AP2 domains [46]. Meanwhile, in *Arabidopsis thaliana* the interaction of *trehalose 6-phosphate* (*T6P*) with *SnRK1* can reduce the phosphorylation of *WRI1* half-life [47], which positively regulates the biosynthesis of fatty acids [47,48].

This process implies a shift of the carbon flow from source tissues into newly established sink tissue and an allocation of carbon to synthesize specific storage molecules useful for the seed. Therefore, it is likely that *WRI1* gene, up-regulated in pistachio “ON” fruits as well as *T6P*, is involved in the sink strength of the pistachio seed by modulating carbon allocation into oil. In our study, the up-regulation of *MADS-box* genes in the “ON” season fruits were evident, implying their involvement in fruit/embryo development and possible hormone/nutrient signaling. *MADS-box* genes can orchestrate different developmental programs including organ senescence and abscission that respond to internal and external signals such as hormones [49,50,51]. Among the *MADS-box* genes, *AGAMOUS*, which was found up-regulated in ON fruits, is directly involved in the activation of the *jasmonic acid* (*JA*) *biosynthesis* gene [51].

Jasmonate (*oxophytodienoate reductase 1*) and salicylic acid-related genes showed opposite expression pattern in ON fruit and bud tissues. These genes were down-regulated in “ON” bud, whereas they were up-regulated in “ON” fruits. In banana, treatments with SA and JA enhanced autophagy-mediated banana resistance due to pathogens attack [52]. Therefore, it would be challenging to further investigate the Jasmonate and salicylic acid crosstalk with hormones (ethylene and ABA, in particular) and reactive oxygen species (ROS) for better understanding their possible role in tailoring of the plant response to the nutritional stress. Jasmonate and salicylic acid-related genes can be also used for developing biomarkers for early detection of stress.

The involvement of hormonal factors in alternate pistachio bearing has been studied by many authors [6,53,54]. The present study showed that different hormones pathways are involved interactively, presumably as a response to signals of degradation and remobilization of carbon and nutrients due to the strength of the developing embryos. Comparing RNA-Seq results from both inflorescence bud [11] and fruit, gibberellin-related genes resulted up-regulated in “ON” fruits and buds while the auxin-related genes are down-regulated in in both. This finds support two recent qualitative analysis conducted by Gündeşli, 2020 [54] and Gündeşli et al., 2020 [55] showing an increase in gibberellins and a decrease in auxins levels in pistachio “ON” organs possibly linked to initial embryo development and an evident correlation with flower bud abscission. It is also noticeable that auxin exerts inhibitory effects on the cytokinin pathway and signaling mechanisms, as reported in different studies [55,56].

In our experiment, most of the cytokinin-related genes in “ON” fruits showed up-regulation, whereas some of these genes showed down-regulation in “ON” inflorescence buds [11]. This can be assumed as a sign of competition between pistachio fruits and inflorescence buds during the “ON” and “OFF” season and indicate the strength of the fruit. Many studies have shown that cytokinin availability is related to plant sink strength under stress [56,57,58]. A possible explanation is that cytokinin may increase the sink capacity by intensifying cell proliferation or regulating sucrolytic enzymes’ activity gaining more photo-assimilates, as demonstrated in grapevine (*Vitis vinifera* L.) [58]. Similar trends showed ABA related genes down-regulated in “ON” buds and up-regulated in “ON” fruits. Thus, a potential role of ABA and cytokinin can be suggested in reinforcing the sink strength of developing embryo/fruits. Furthermore, sink competition due to lack of nutrients can induce oxidative stress and ROS accumulation leading to autophagy in inflorescence buds [11]. Nevertheless, a negative correlation between polyamines and bud abscission has been found in pistachio trees [8,10,11,59] Thus, the competition between polyamines and ethylene pathways for *S*-adenosyl methionine can result in a mechanism that can modulate physiological events, including senescence and inflorescence bud abscission. 

## 5. Conclusions

Our findings indicate that the main leading causes of premature inflorescence bud abscission are the shortage of nutrients and that crosstalk among sugar and various hormone relate genes, e.g., ABA, cytokinin and ethylene, occur regulating sink-source development, interaction, and organ fate within the same individual branches. Hormone applications may mitigate the phenomenon; however, accurate management of resources like carbohydrates and mineral elements directly or indirectly linked to the mechanism can modulate the rate of alternating production. 

In the future, it would be interesting to develop potentially useful biomarkers indicating nutritional stress (e.g., *callose synthase 5*, *trehalose-6-phosphate synthase*, *NAD(P)-linked oxidoreductase* and *MIOX2*, Jasmonate, and salicylic acid-related genes) and to ascertain whether the molecular mechanisms described can also explain the premature fall of other organs (flowers and flower parts, fruitlets, leaves) in other fruit species when they are subjected to nutritional stress. Furthermore, it would be interesting to check if exogenous trehalose application can mitigate the phenomenon. The present work showed how “omic” studies could be effectively used to identify molecular gene regulatory networks occurring in plants involved in physiological responses to hormone dysregulation and environmental stresses such as previously found for other crops [59,60,61]. As concern cultivation practices, it is certainly necessary to prevent the tree from encountering biotic and abiotic stresses that can generally exacerbate the phenomenon, to set up proper irrigation and precision pruning practices to balance the sink/source equilibrium and to enhance photosynthesis, and to employ vigorous rootstock to ensure good water and carbohydrate resource status. At the same time, the finding of putative biomarkers in the future may lead to plan precision farming practices to reduce and balance the alternate bearing phenomenon in the context of more advanced and profitable pistachio farming.

## Figures and Tables

**Table 1:** Bud drops percentage during the season in bearing and non-bearing pistachio branches (SE = standard error).

	June (%)	SE	July (%)	SE	September (%)	SE
**ON**	7.4	±4	54.7	±10	78.3	±11
**OFF**	0	±0	25.4	±3	31	±5

**Table 2:** The table provides the number of total genes, up- and down-regulated genes in fruits during the current year non-bearing shoot (“OFF”) and bearing shoots (“ON”).

Comparison	Differentially Expressed Genes	Up-Regulated	Down-Regulated
June “OFF” vs. June “ON”	1536	702	834
July “OFF” vs. July “ON”	950	482	468

## Data Availability

The sequencing data was stored in the Sequence Read Archive (SRA) of the National Center for Biotechnology Information (NCBI). The SRA accession Number PRJNA623387.

## Supplementary Materials

The following are available online at https://www.mdpi.com/article/10.3390/genes13010060/s1. Table S1: The table provides the details of raw and pre-processed data. Table S2: The table shows the overall assembly and evaluation statistics. Table S3: The table shows DEGs enciphering for photosynthetic pathway in “OFF” fruit vs. “ON” fruit in June. Figure S1: The figure shows the bearing branches and fruit/embryo stages in June and July “ON”. Figure S2: The figure shows the “ON” and “OFF” branches. Figure S3: The figure shows the workflow used for the *Pistachio* de novo discovery and analysis. Figure S4: The dendrogram plot showing the clustering patterns of the samples. Figure S5: Figure shows transcription factors among the July “OFF” vs. July “ON” fruit comparison. The y-axis indicates the log2 FC value. The bar represents the differentially expressed genes in July “OFF” fruits relative to July “ON” fruits. Figure S6: Figure shows hormone metabolism in Pistachio among the July “OFF” vs. July “ON” fruit comparison. Figure S7: Figure shows polyamine biosynthesis pathway in pistachio fruit among the June “OFF” vs. June “ON” comparison. The color scale indicates the log2 FC value. Red represents down-regulation and blue represents up-regulation in June “OFF” relative to June “ON” buds. Arrows indicate the effects on PA levels driven by overexpression of different PA biosynthesis genes. Figure S8: The comparative study on starch metabolism of the July “OFF” vs. July “ON” fruits.
