# Supplementary figures and images for: Transcriptomic Analysis of the Pistacia vera (L.) Fruits Enable the Identification of Genes and Hormone-Related Gene Linked to Inflorescence Bud Abscission
Source: Genes (Basel). 2021 Dec 27;13(1):60. doi: 10.3390/genes13010060 (PMC8774834; doi:10.3390/genes13010060)

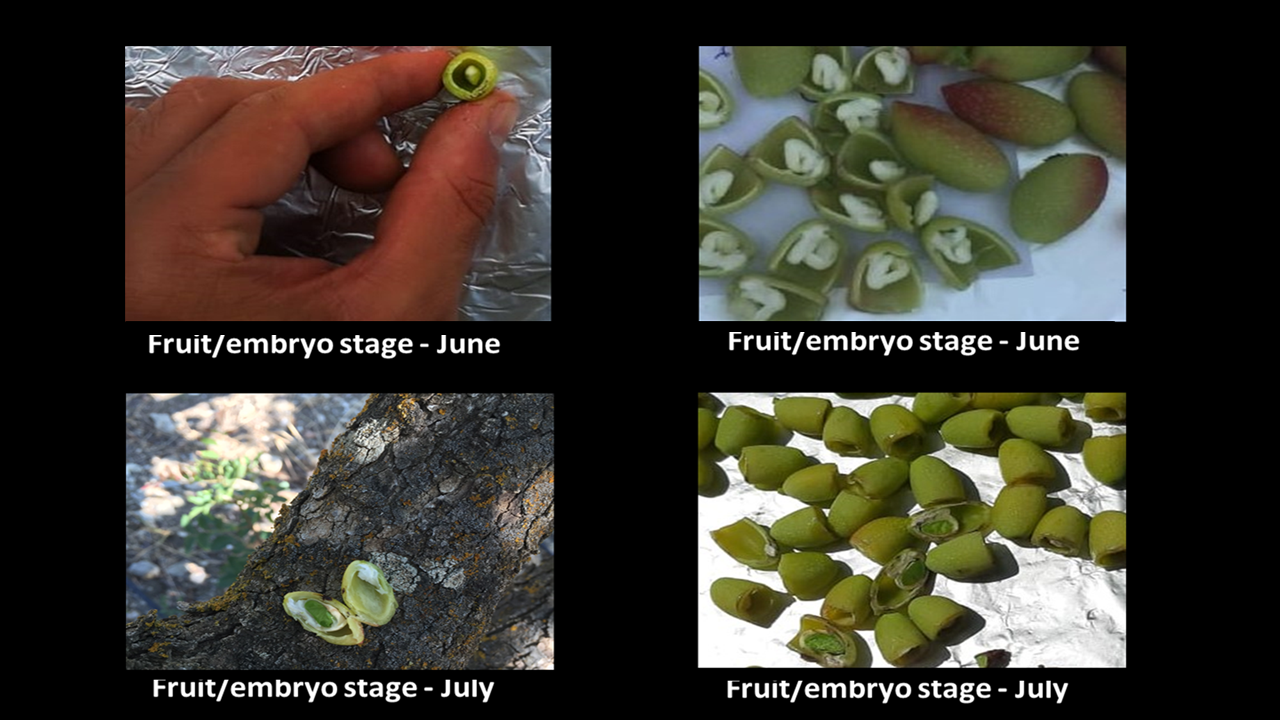

Supplement: Supplementary file 1 [file genes-13-00060-s001.zip › Figure S1.tif]

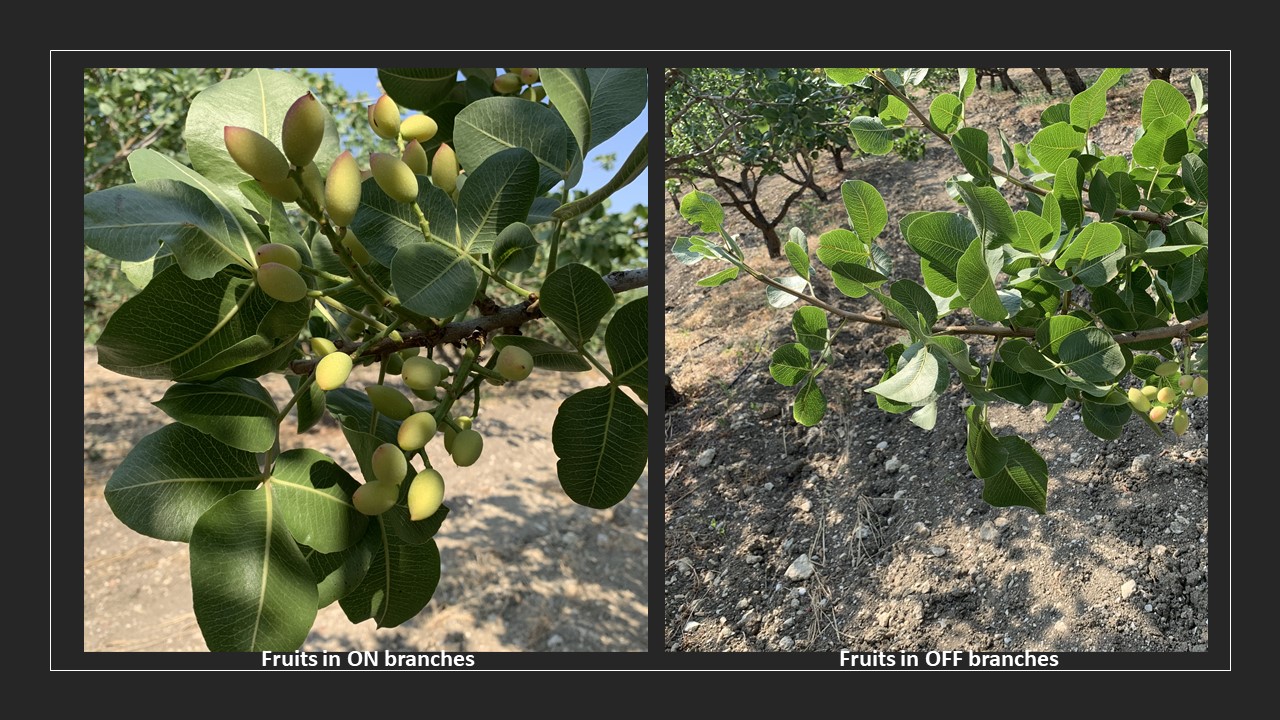

Supplement: Supplementary file 1 [file genes-13-00060-s001.zip › Figure S2.jpg]

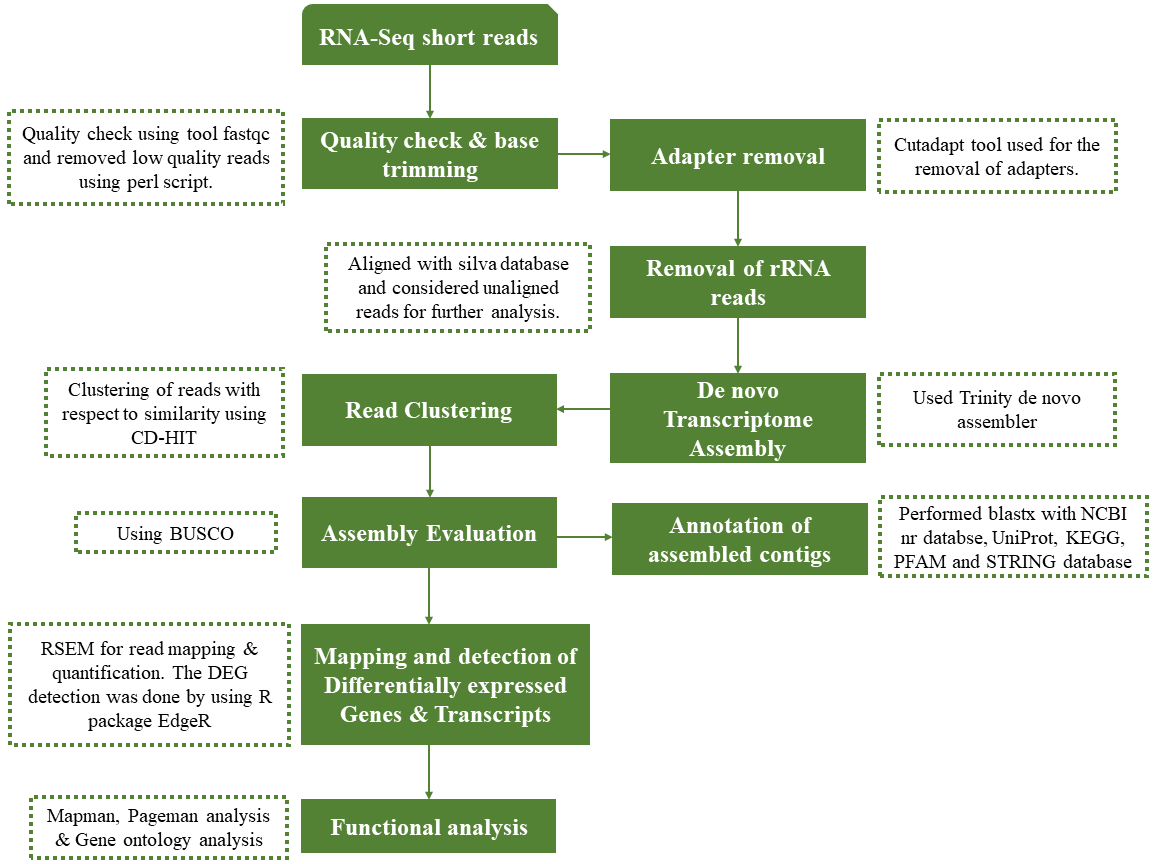

Supplement: Supplementary file 1 [file genes-13-00060-s001.zip › Figure S3.tif]

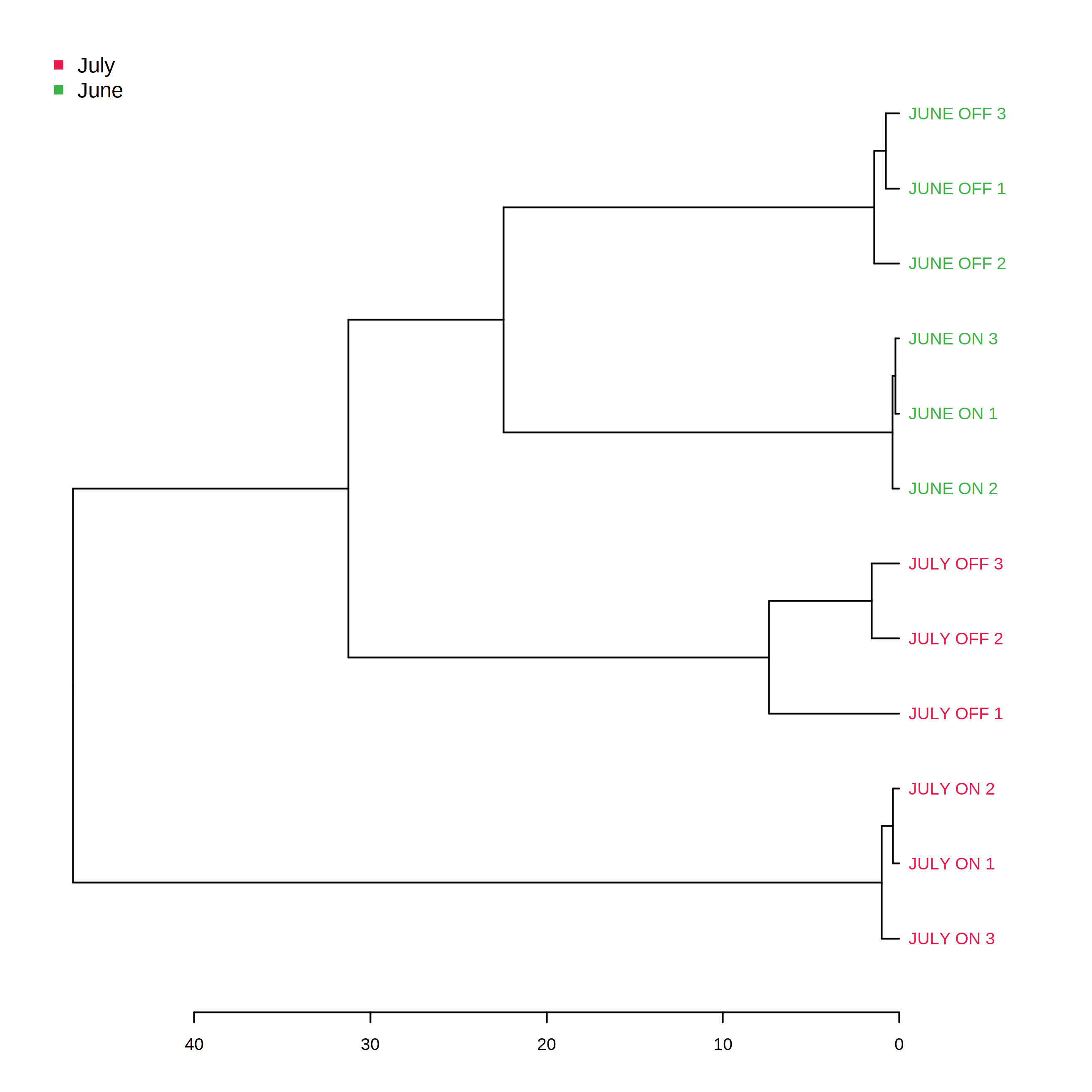

Supplement: Supplementary file 1 [file genes-13-00060-s001.zip › Figure S4.tiff]

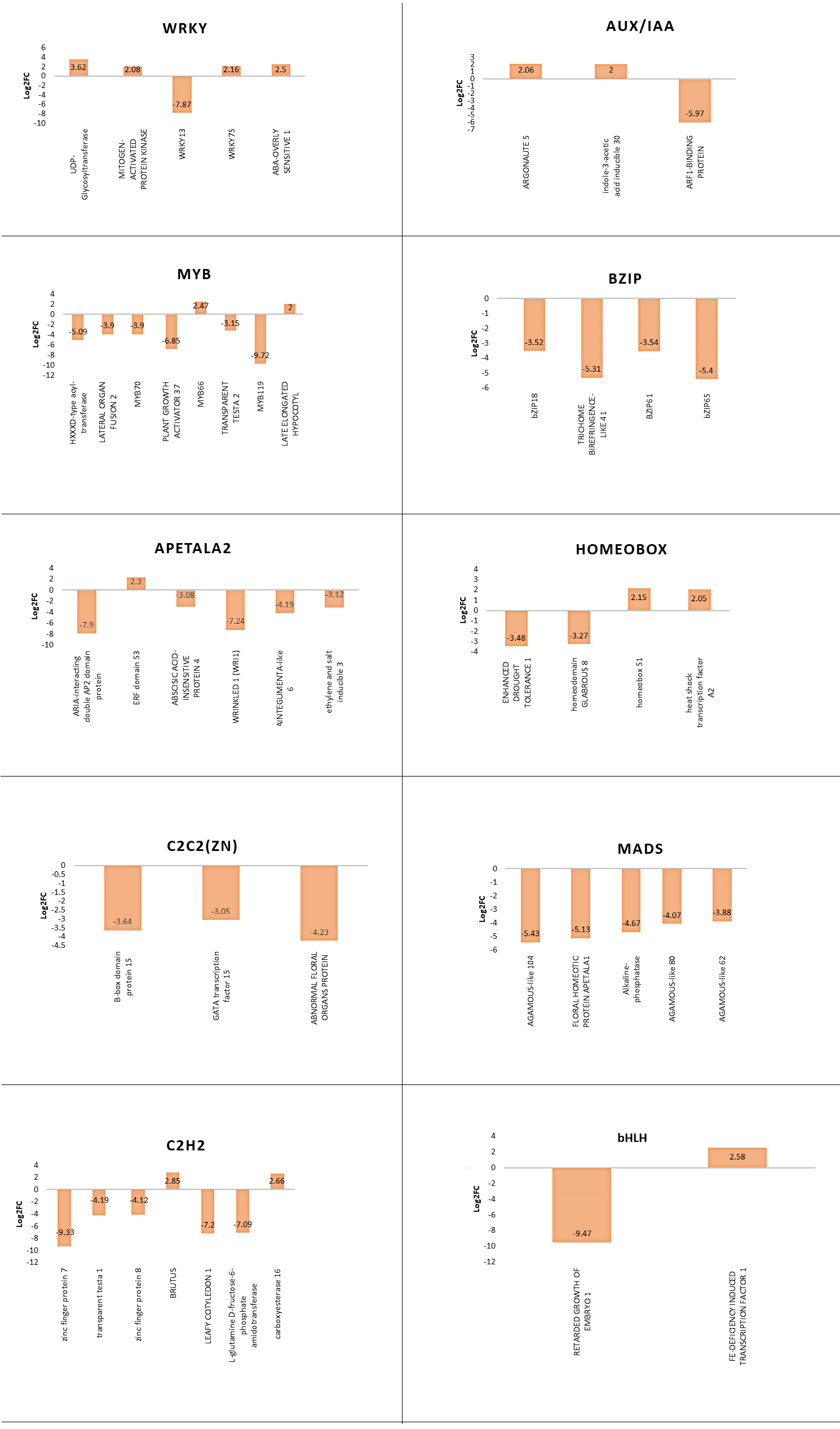

Supplement: Supplementary file 1 [file genes-13-00060-s001.zip › Figure S5.tif]

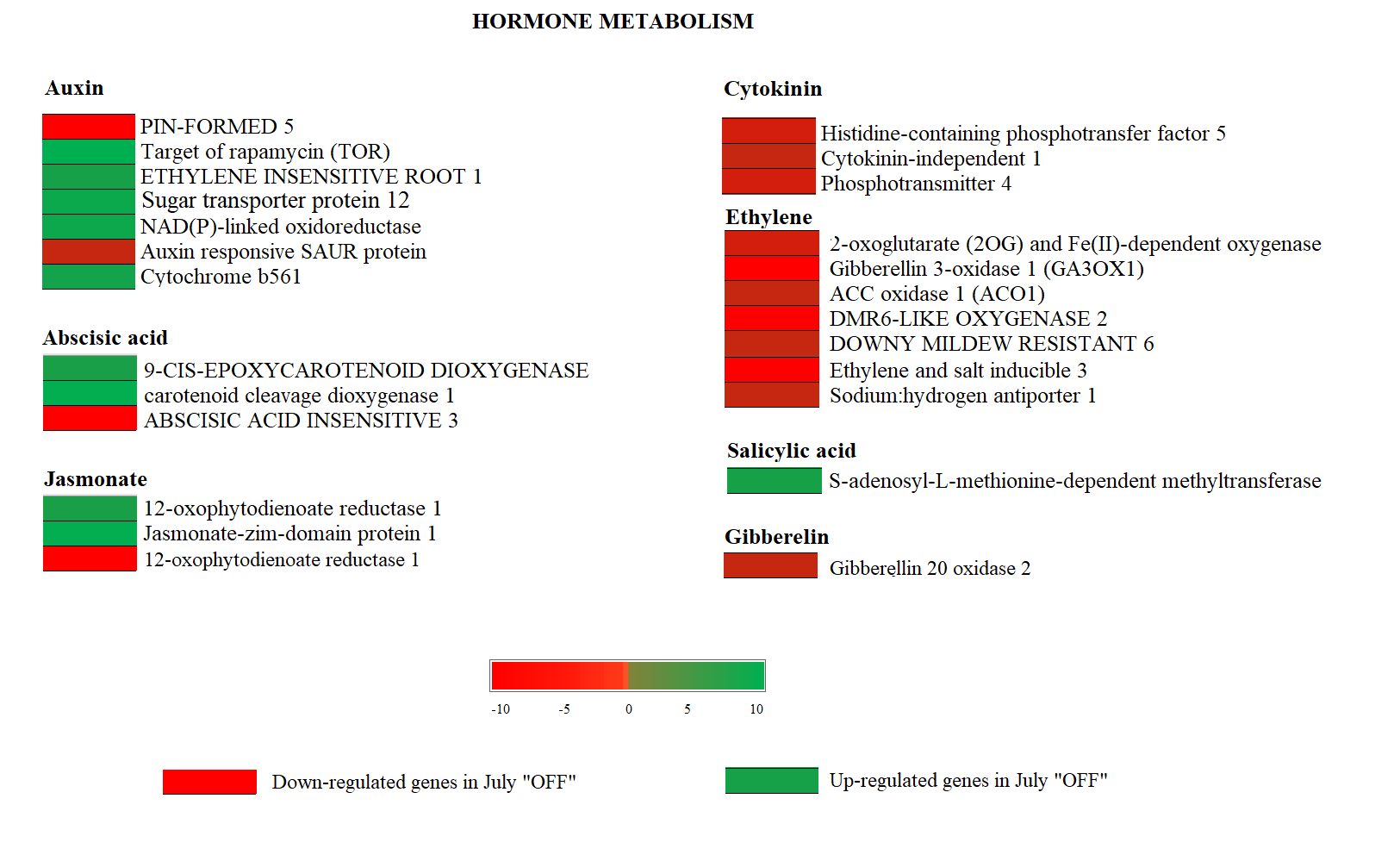

Supplement: Supplementary file 1 [file genes-13-00060-s001.zip › Figure S6.png]

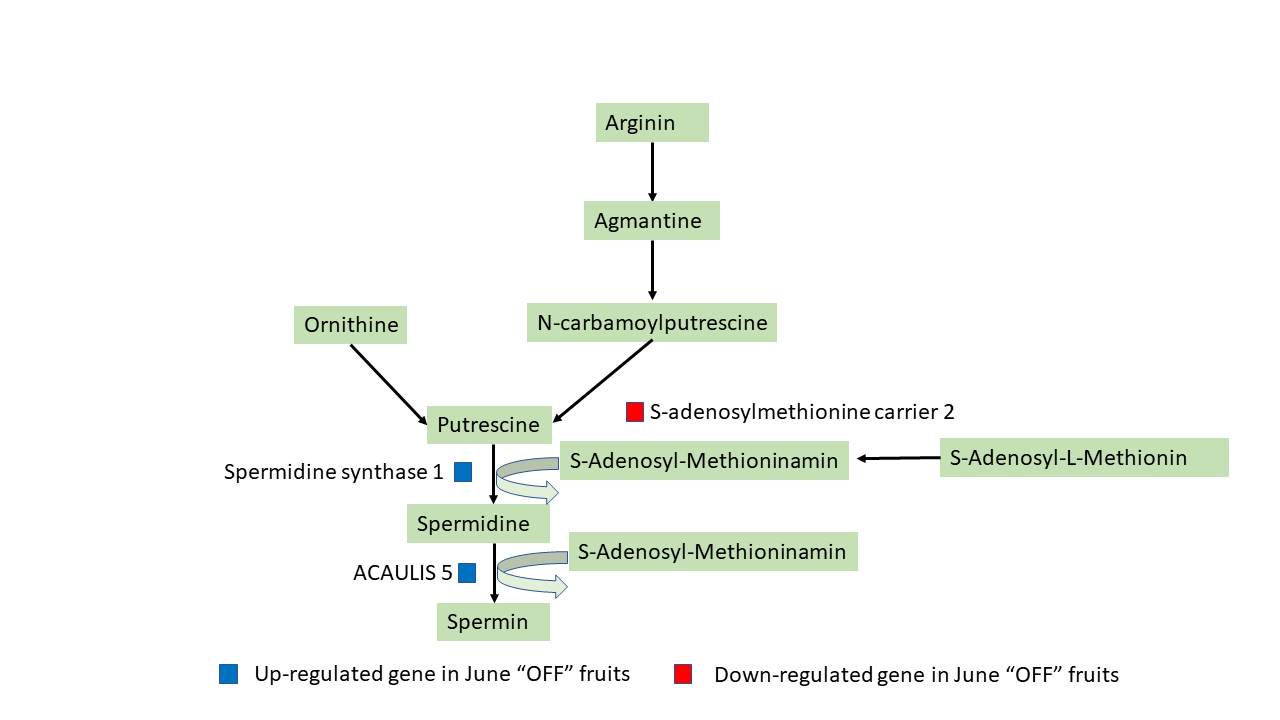

Supplement: Supplementary file 1 [file genes-13-00060-s001.zip › Figure S7.tif]

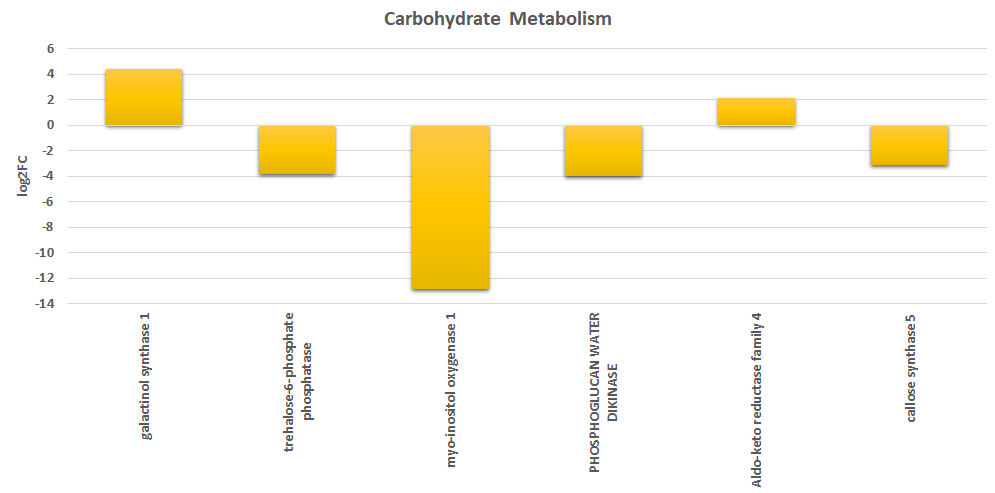

Supplement: Supplementary file 1 [file genes-13-00060-s001.zip › Figure S8.tif]
